# RegNetwork: an integrated database of transcriptional and post-transcriptional regulatory networks in human and mouse

**DOI:** 10.1093/database/bav095

**Published:** 2015-09-30

**Authors:** Zhi-Ping Liu, Canglin Wu, Hongyu Miao, Hulin Wu

**Affiliations:** ^1^Department of Biomedical Engineering, School of Control Science and Engineering, Shandong University, Jinan, Shandong 250061, China and; ^2^Department of Biostatistics, School of Public Health, University of Texas Health Science Center at Houston, Houston, TX 77030, USA

## Abstract

Transcriptional and post-transcriptional regulation of gene expression is of fundamental importance to numerous biological processes. Nowadays, an increasing amount of gene regulatory relationships have been documented in various databases and literature. However, to more efficiently exploit such knowledge for biomedical research and applications, it is necessary to construct a genome-wide regulatory network database to integrate the information on gene regulatory relationships that are widely scattered in many different places. Therefore, in this work, we build a knowledge-based database, named ‘RegNetwork’, of gene regulatory networks for human and mouse by collecting and integrating the documented regulatory interactions among transcription factors (TFs), microRNAs (miRNAs) and target genes from 25 selected databases. Moreover, we also inferred and incorporated potential regulatory relationships based on transcription factor binding site (TFBS) motifs into RegNetwork. As a result, RegNetwork contains a comprehensive set of experimentally observed or predicted transcriptional and post-transcriptional regulatory relationships, and the database framework is flexibly designed for potential extensions to include gene regulatory networks for other organisms in the future. Based on RegNetwork, we characterized the statistical and topological properties of genome-wide regulatory networks for human and mouse, we also extracted and interpreted simple yet important network motifs that involve the interplays between TF-miRNA and their targets. In summary, RegNetwork provides an integrated resource on the prior information for gene regulatory relationships, and it enables us to further investigate context-specific transcriptional and post-transcriptional regulatory interactions based on domain-specific experimental data.

**Database URL**: http://www.regnetworkweb.org

## Introduction

Gene regulatory events play crucial roles in a variety of physiological and developmental processes in a cell, in which macromolecules such as genes, RNAs and proteins are coordinated to orchestrate operative responses under different conditions (1). Therefore, substantial efforts have been made to reveal gene regulatory network structures from transcriptomic profiling datasets generated by, e.g. microarray (2), ChIP-Seq (3) and RNA-Seq (4). Although a number of data-driven reverse engineering techniques were previously proposed to identify regulatory relationships between regulators and their targets [e.g. TFs and downstream genes (5)], the low accuracy of these existing methods due to the curse of dimensionality significantly limits their applications in practice (6). However, several recent studies suggested a promising alternative for identifying regulatory network structures by combining the high-throughput transcriptomic profiling data with the prior knowledge on known or predicted regulatory relationships available in various databases and literature (7–9). For instance, the framework in (9) can significantly improve the accuracy of regulatory relationship identification by appropriately incorporating prior knowledge into the transcriptomic profiling data. Also, the results from several other independent studies suggest that the incorporation of prior knowledge can help to better identify the context-specific regulatory interactions corresponding to certain phenotypes (7–12). It is thus of paramount interest to collect, organize and share such prior information with the related communities for future biomedical research and practice.

Prior knowledge on gene regulatory relationships from multiple sources (e.g. genomic context, conserved gene co-expression, knockout or high-throughput experiment) spreads out in various databases and literature. It is desirable to develop a unified database and provide users with the necessary tools for information access or retrieval. However, only limited efforts such as RegulonDB for *Escherichia coli* (13) have been previously made towards this goal, and the works on the genome-wide regulatory relationships for other species are still lacking so far. Considering the overwhelming importance of human and mouse in biomedical studies, we build a database of genome-wide regulatory relationships for the two species. It should be noted that, besides the experimentally observed or discovered regulatory relationships curated in public databases such as TRED (14) and KEGG (15), the TF binding site (TFBS) information for TF–gene regulatory interaction potentials (16, 17) can also be used to predict new transcriptional regulatory relationships between TFs and genes by matching the binding motifs in DNA sequences. Thus, such predictions based on TFBS are also integrated into our database to provide a more comprehensive landscape of gene regulations. Moreover, to include post- transcriptional regulatory relationships in the database, we also consider miRNAs, which are small non-coding RNA molecules (∼22 nucleotides) found in various organisms (18) and ubiquitously perform crucial roles in post- transcriptional regulation of gene expression by binding to the 3′ untranslated region of mRNA (19).

Although there exist many computational methods for deciphering the transcriptional regulatory interactions between TFs and genes, the integrative analysis considering both TF and miRNA as regulators is still very limited due to the lack of a ready-to-use regulatory network database (20). In recognition of such an emerging need, here we build a comprehensive database for genome-wide regulatory networks at both transcriptional and post- transcriptional levels for human and mouse by integrating the documented regulatory relationships from 25 databases. RegNetwork can be freely accessed at http://www.regnetworkweb.org.

## Materials and methods

### Data sources

**Figure 1.**
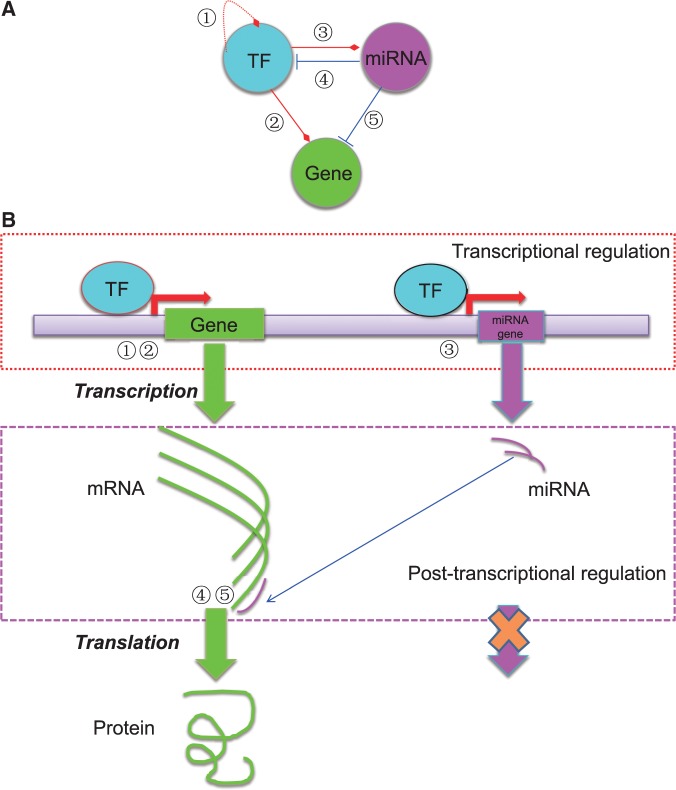
The basic regulatory circuit involving TF, miRNA and target gene (**A**) and the schematic illustration of the mechanisms of transcriptional and post-transcriptional regulation of gene expression (**B**). In total, five types of regulatory relationships are considered among TF, miRNA and target gene.

Both transcriptional and post-transcriptional regulatory relationships are important, we thus consider both TFs and miRNAs as regulators. Figure 1 shows a basic regulatory circuit involving TF, miRNA and target gene, as well as the essential steps of transcriptional and post- transcriptional regulation of gene expression. Note that the miRNA component is usually missing in most of the previous studies on reverse engineering of gene regulatory networks. However, given its important role in the post-transcriptional regulatory process (18), we believe that it is necessary to include miRNAs in RegNetwork.

As shown in Figure 1, five types of regulatory relationships among TFs, miRNAs and target genes are considered in the regulatory network. More specifically, for transcriptional regulatory relationships, ‘TF-TF’ (①) and ‘TF-gene’ (②) interactions are considered; for post-transcriptional regulatory relationships, the curated and predicted ‘miRNA-gene’ interactions (⑤) are considered; for interplays between regulators, ‘TF-miRNA’ (③) and ‘miRNA-TF’ (④) are included. A number of databases contain regulatory information for human and mouse, from which we collect the relevant information and data (e.g. TFs, miRNAs, TFBS motifs, genes and their annotations). Table 1 lists the databases we used to build the RegNetwork.

**Table 1.** The databases used to build the RegNetwork database by collecting knowledge on gene regulatory relationships in human and mouse

Database	Description	Species	Website	Reference	Version/access date
*BioGrid*	BioGRID is an online interaction repository with data compiled through comprehensive curation efforts	Mouse	http://thebiogrid.org/	(21)	Version 3.2.100
*Ensembl*	Ensembl is to provide a centralized resource for geneticists, molecular biologists and other researchers studying the genomes of our own species and other vertebrates and model organisms	Human and mouse	http://www.ensembl.org	(22)	Release 71 (March 2013)
FANTOM	Functional Annotation Of Mammalian genome and is an international research consortium to assign functional annotations to the full-length complementary DNAs (cDNAs)	Human and mouse	http://fantom.gsc. riken.jp/	(23)	5 March 2010
GenBank	A comprehensive database developed by NCBI, NIH, which contains publicly available nucleotide sequences for more than 250 00 formally described species	Human and mouse	http://www.ncbi.nlm.nih. gov/genbank/	(24)	14 August 2012
*HPRD*	HPRD is a curated human protein-protein interaction database	Human	http://www.hprd.org	(25)	Release 9
*IntAct*	IntAct is a database system of molecular interaction data. All interactions are derived from literature curation or direct user submissions	Mouse	http://www.ebi.ac.uk/intact/	(26)	16 October 2012
JASPAR	An open-access database of annotated, matrix-based transcription factor binding site (TFBS) profiles for multicellular eukaryotes	Human and mouse	http://jaspar.genereg.net/	(17)	12 October 2009
*KEGG*	KEGG is a widely used pathway database resource for understanding high-level linkage functions and utilities of biological system	Human and mouse	http://www.genome.jp/kegg/	(15)	5 December 2012
Liftover	A UCSC tool converts genome coordinates and genome annotation files between assemblies	Mouse	http://genome.ucsc.edu/cgi-bin/hgLiftOver	(27)	7 March 2012
MicroCosm	MicroCosm Targets (formerly miRBase Targets) is a web resource containing computationally predicted targets for microRNAs across many species	Human and mouse	http://www.ebi.ac.uk/enright-srv/microcosm/htdocs/targets/v5/	(28)	Version v5
*MicroT*	DIANA-microT is a combined computational- experimental approach predicts mouse microRNA targets	Human and mouse	http://www.microrna.gr/microT	(29)	Version v3.0
*miRanda*	miRanda is a miRNA target prediction method based on dynamic programming algorithm	Human and mouse	http://www.microrna. org/	(30)	Release August 2010
*miRBase*	miRBase database is a searchable database of published miRNA sequences and annotation	Human and mouse	http://www.mirbase.org/	(31)	Release 18
*miRecords*	miRecords is a resource for animal miRNA-target interactions. The validated targets component is used, which is a large, high-quality database of experimentally validated miRNA targets	Human and mouse	http://miRecords.umn. edu/miRecords	(32)	25 November 2010
*miRTarBase*	miRTarBase is a database which curates experimentally validated microRNA-target interactions	Human and mouse	http://miRTarBase.mbc.nctu.edu.tw/	(33)	Release 2.5 (October 2011)
*PicTar*	PicTar is a computational method for identifying common targets of microRNAs	Human and mouse	http://pictar.mdc-berlin.de/	(34)	26 March 2007
RefSeq	RefSeq provides a non-redundant collection of sequences representing genomic data, transcripts and proteins	Human and mouse	http://www.ncbi.nlm.nih. gov/refseq/	(35)	19 May2013
*STRING*	STRING is a database of known and predicted protein interactions	Mouse	http://www.string-db.org	(36)	Version 9.05
*Tarbase*	Tarbase collectes available miRNA targets derived from all contemporary experimental techniques (gene specific and high-throughput)	Human and mouse	http://www.microrna.gr/tarbase	(37)	Version 5.0
*TargetScan*	TargetScan is an algorithm to predict biological targets of miRNAs by searching for the presence of conserved 8mer and 7mer sites that match the seed region of each miRNA	Human and mouse	http://www.targetscan .org/	(38)	Release 5.2
TRANSFAC	Transfac database is a manually curated database of eukaryotic transcription factors, their genomic binding sites (TFBS) and DNA binding profiles	Human and mouse	http://www.gene-regulati on.com/pub/databases.html	(16)	TRANSFAC 7.0
*TransmiR*	TransmiR is a transcription factor-microRNA regulation database	Human and mouse	http://202.38.126.151/hmdd/mirna/tf/	(39)	Version 1.2
*TRED*	Transcriptional Regulatory Element Database (TRED) is an integrated repository repository for both cis- and trans- regulatory elements in mammals. It contains the curated regulations between TF and target gene	Human and mouse	http://rulai.cshl.edu/TRED/	(40)	12 February 2012
UniProt	UniProt is a catalog of information on proteins and it is a central repository of protein sequence and function	Human and mouse	http://www.uniprot.org/	(41)	Release July2012
*UCSC*	The University of California, Santa Cruz Genome Browser is a database of genomic sequence and annotation data for a wide variety of organisms	Human and mouse	http://genome.ucsc.edu	(27)	mm10, GRCm38 (December 2011)

The ‘Species’ column shows whether the information in a database is available for human, mouse or both. Twenty-five databases are used to build the RegNetwork and they are ordered alphabetically here, among which 17 of these databases in italic contain the regulatory relationships, and the rest provide other necessary information (e.g. annotations) for the database construction.

### Regulatory relationship curation and prediction

**Figure 2.**
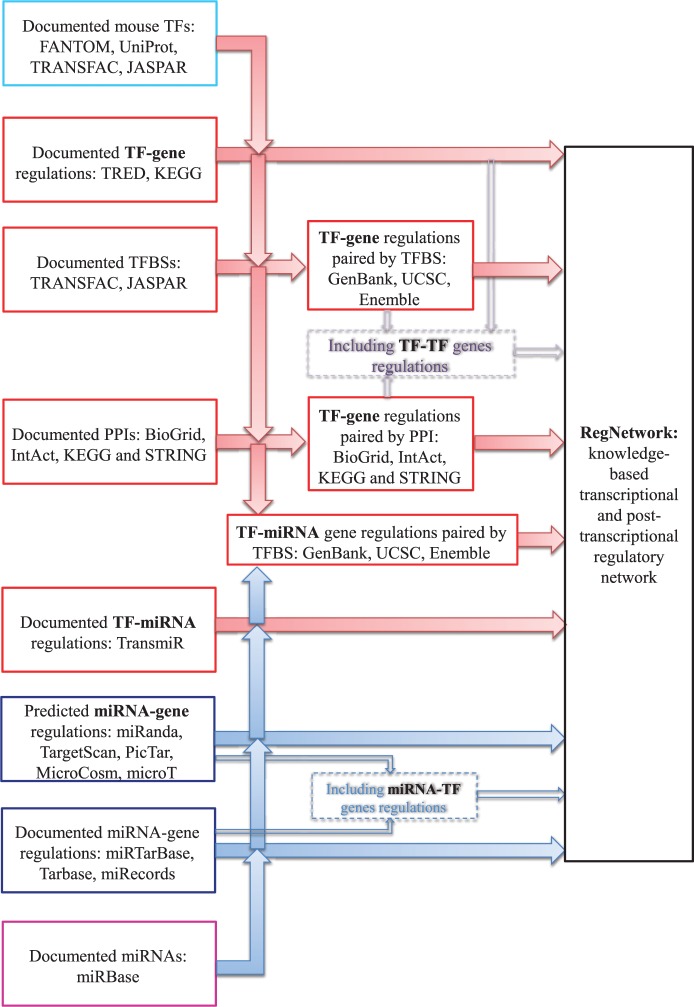
The flowchart for RegNetwork construction.

Figure 2 illustrates how the databases listed in Table 1 are used to construct the RegNetwork, and the same procedure in forms of tested computer code is performed for human and mouse, respectively.

**Figure 3.**
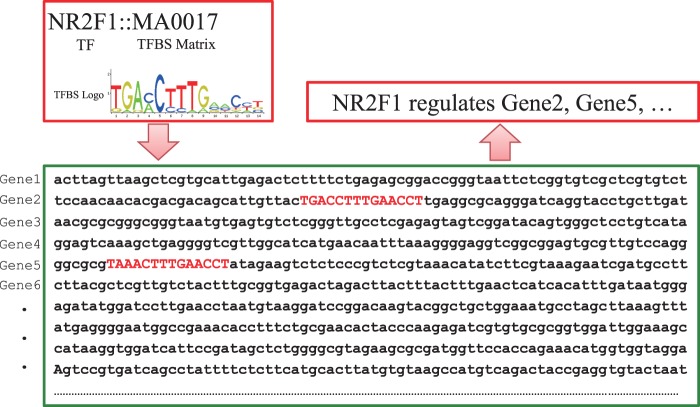
Schematic illustration of pairing TF and genes by TFBSs. When the documented TFBS ‘MA0017’ is found in the promoter regions of ‘Gene2’ and ‘Gene 5’, TF NR2F1 is predicted to have a potential to regulate the two genes accordingly.

More specifically, for transcriptional regulatory relationships, we first compile a list of TFs for human and mouse, respectively, from FANTOM (23), UniProt (41), TRANSFAC (16) and JASPAR (17). Then, the ‘TF-gene’ interactions documented in TRED and KEGG are directly deposited in RegNetwork. Moreover, we predict the potential ‘TF-gene’ interactions from the documented TF binding site (TFBS) motifs in TRANSFAC and JASPAR. Since TFs regulate the target genes by binding to these experimentally identified TFBSs, we pair the TFs and genes by searching the promoter regions from the 5 kb upstream to 1 kb downstream of the transcription start site (TSS) for RefSeq (35) genes. Figure 3 illustrates the basic idea of how to pair a TF with the potential target genes via TFBS. As an example, TF ‘NR2F1’ has a known TFBS ‘MA0017’, which is represented by a position weighted matrix, and the sequence logo at the top-left corner in Figure 3 shows its nucleotides composition. Screening the promoter regions in the whole genome of human and mouse for this TFBS, the genes containing ‘MA0017’ in their promoter regions are thus identified as the potential targets of ‘NR2F1’. In general, we retrieve the information of TFBS conservation tracks from the UCSC Genome Browser (27) and Ensembl (22) database. Specifically, UCSC’s tfbsConsSites table contains the location and score of TFBS conserved in the human/mouse sequence alignment results. A binding site is considered to be conserved across the alignment results if its score is no less than the threshold score. The score and the threshold are computed with the TRANSFAC matrices by the TFLOC program (27). Since the UCSC only implements the TFBS conservation tracks in human genome, we map the TFBS conservation information to mouse genome by employing the LiftOver (27) tool of UCSC. Similarly, Ensemble’s MotifFeatures.gff table contains the alignment information for the TFBS element matrix documented in JASPAR [by MOODS software (42)] for human and mouse. The chromosomal coordinates of TFBSs can be used to identify their corresponding genes and the potential regulatory relationships between TFs and genes can then be established. To include as many TFs and their interaction targets as possible in our database, we also consider and include protein–protein interactions (PPIs) in RegNetwork. We retrieve the PPI pairs that contain at least one TF from HPRD (25), BioGrid (21), IntAct (26), KEGG (15) and STRING (36). The functional linkages between TF and its interacting partners indicate putative gene regulations. Obviously, when a TF regulates the expression of its own gene, the ‘TF-TF’ self-regulations are also identified. To be consistent in this process, TFs and genes are represented using their corresponding NCBI Entrez IDs and official symbols (24).

For post-transcriptional regulations, the experimentally validated ‘miRNA–gene’ pairs in human and mouse from miRTarBase (33), TarBase (37) and miRecords (32) are directly deposited in RegNetwork. Then, the predicted ‘miRNA–gene’ interactions by one of the five representative algorithms, i.e. miRanda (30), TargetScan (38), PicTar (34), MicroCosm (28) and micorT (29), are included. Similarly, the documented ‘miRNA–TF’ genes regulatory relationships are directly deposited into RegNetwork.

The documented ‘TF–miRNA’ regulatory relationships in TransmiR (39) are also directly imported into RegNetwork. Then, the potential interactions between TFs and miRNA-encoding genes are predicted based on TFBS information using the similar method for potential ‘TF–gene’ interactions as described above. In such a way, the documented and putative regulatory pairs are both included in RegNetwork. For certain pairs of genes, the regulator (or target) gene in one database may be labeled as target (or regulator) gene in another database. We merged such results and thus the interactions between these pairs of genes can be bidirectional. That is, we used an ‘inclusive’ principle to deal with the inconsistency between the databases. At the same time, we also provide a link to the original databases for users to check the detailed information regarding the inconsistency and decide which result they will believe and use for a particular case. Finally, we added the degree of confidence for each of the regulatory interactions by using a three-level labeling approach (i.e. a ‘high’, ‘medium’ or ‘low’ confidence). More specifically, the experiment-validated regulations are tagged with the label ‘high confidence’, the predictions made by only one algorithm/method are tagged with ‘low confidence’, and the rest are tagged with ‘medium confidence’.

### Database implementation and web user interface design

We have developed a web tool of RegNetwork for users to query and download the regulatory relationships and networks. RegNetwork is implemented in Java, JavaScript and Python together with the PostgreSQL database. All raw data ETL (Extract, Transfer and Load) are carried out with Python scripts on the back end. The frontend interface is developed using JSP and JavaScript.

**Figure 4.**
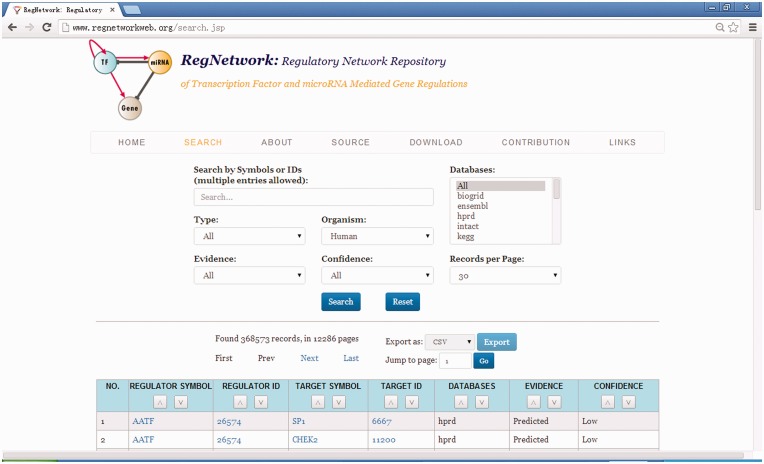
The web user interface of RegNetwork.

Figure 4 shows the web user interface of RegNetwork. The regulatory relationship can be searched by various types of components (i.e. by TF, miRNA or gene in the regulatory networks), by databases and/or by species (human or mouse). The interface also provides users the option to query transcriptional only, post-transcriptional only or both relationships to further refine the search. It also allows users, while querying RegNetwork, to specify and constraint the original databases where the regulatory relationships are derived from. The query results can be exported as a CSV file. The users can employ some tools such as Sig2BioPax (43) to convert the regulations into the BioPAX Level 3 format (44). Also, the full datasets are made available for users to download for further analyses.

## Results and discussion

### Regulatory networks in human and mouse

By integrating the experimental, inferred or predicted regulatory interactions among TFs, miRNAs and genes from a variety of sources, we developed a database named RegNetwork as a comprehensive repository for genome-wide regulatory networks in human and mouse. RegNetwork contains both transcriptional and post- transcriptional regulatory relationships, and the interplays between TF/miRNA and their targets can then be easily retrieved from the database. In addition, the data source information for the regulatory relationships can also be retrieved from RegNetwork. As of June 2015, the basic statistics of the regulatory networks in RegNetwork are calculated and listed in Table 2. Specifically, the human regulatory network contains 23 079 nodes and 369 277 edges, consisting of 1456 TFs, 1904 miRNAs and 19 719 target genes; and the mouse regulatory network contains 20 738 nodes and 323 636 edges, consisting of 1328 TFs, 1290 miRNAs and 18 120 target genes. For details of how to use expression data to identify subnetworks from the background network under specific conditions, the interested reader is referred to Liu *et al.* (9).

**Table 2.** The basic statistics of the regulatory networks of human and mouse in RegNetwork

Element	Description	Number	
		Human	Mouse
Node	All nodes included in the regulatory network	23 079	20 738
Edge	All regulatory relationships included in the regulatory network	369 277	323 636
TF	The documented TFs included in the regulatory network	1456	1328
miRNA	The miRNAs included in the regulatory network	1904	1290
Gene	The target genes included in the regulatory network	19 719	18 120
TF–gene	The ‘TF–gene’ regulations included in the regulatory network	149 841	94 876
TF–TF	The ‘TF’–‘TF gene’ self-regulations included the regulatory network	361	129
TF–miRNA	The ‘TF–miRNA gene’ regulations included in the regulatory network	21 744	25 574
miRNA–gene	The ‘miRNA–target gene’ regulations included in the regulatory network	171 477	176 512
miRNA–TF	The ‘miRNA–TF gene’ regulations included in the regulatory network	25 854	26 545

### Network analysis

Real biological networks such as gene regulatory networks and protein–protein interaction networks are different from random networks (45) in terms of certain network properties like characteristic path length and node degree distribution (40, 46). Therefore, network feature analysis allows us to assess whether a network is random or not. Some network feature indices for the established regulatory network for human and mouse from RegNetwork are summarized in Table 3. Particularly, the clustering coefficients of the established regulatory networks in human and mouse are 0.118 and 0.101, respectively, which are much higher than that of random networks of a comparable size (∼$1.5\times 10^{-5}$) (45). Moreover, the characteristic path lengths of the regulatory networks in human and mouse are 3.200 and 3.229, respectively, which are comparatively small, and thus suggest a quick propagation of regulatory information in a non-random manner. All other network topological properties also suggest that the established regulatory networks for human and mouse are different from random networks (45, 46).

**Table 3.** Selected measures in the established regulatory networks for human and mouse

Parameter	Value	
	Human	Mouse
Clustering coefficient	0.118	0.101
Connected components	3	1
Network diameter	8	8
Shortest paths	42 727 382	36 743 196
Characteristic path length	3.200	3.229
Average number of neighbors	31.391	30.548

The definitions of these measures are the same as in Refs. (43, 45).

**Figure 5.**
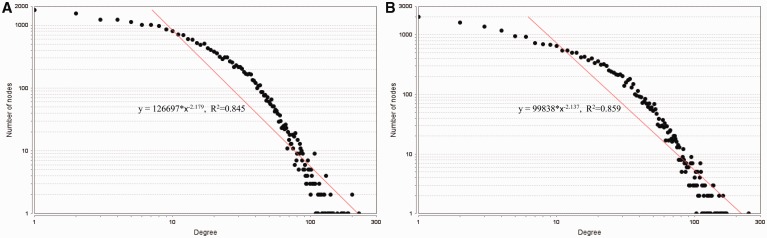
The node degree distributions of the established regulatory networks in human (**A**) and mouse (**B**). A power law distribution in the form of $y=\alpha \cdot x^{-\gamma }$ is fitted in each subfigure, respectively. The results show that the node degrees satisfy the power-law distribution, i.e. $y=\alpha \cdot x^{-\gamma }=126697\cdot x^{-2.179}$, $R^{2}=0.845$ in human, $y=\alpha \cdot x^{-\gamma }=\text{99838}\cdot x^{-2.137}$, $R^{2}=0.859$ in mouse.

Second, the node degrees of the established networks are calculated and found to satisfy the power law distributions as shown in Figure 5. Fitting the power law model $y=\alpha \cdot x^{-\gamma }$, where y denotes the number of nodes and x denotes the node degree, we obtain $\hat{\gamma }=2.179$ for the human regulatory network and $\hat{\gamma }=2.137$ for the mouse regulatory network. Since $2\leq \hat{\gamma }\leq 3$, our background networks are scale-free (45, 46). The network parameters provide evidence that our integrated regulatory networks are different from randomly generated networks. Notice that we employ the definition of random network in (45). A formal and rigorous comparison between the large human/mouse networks derived from our RegNetwork and the corresponding random networks require the use of computing-intensive Monte Carlo approaches, which is beyond the score of this paper.

### Interplays among TF, miRNA and gene

**Figure 6.**
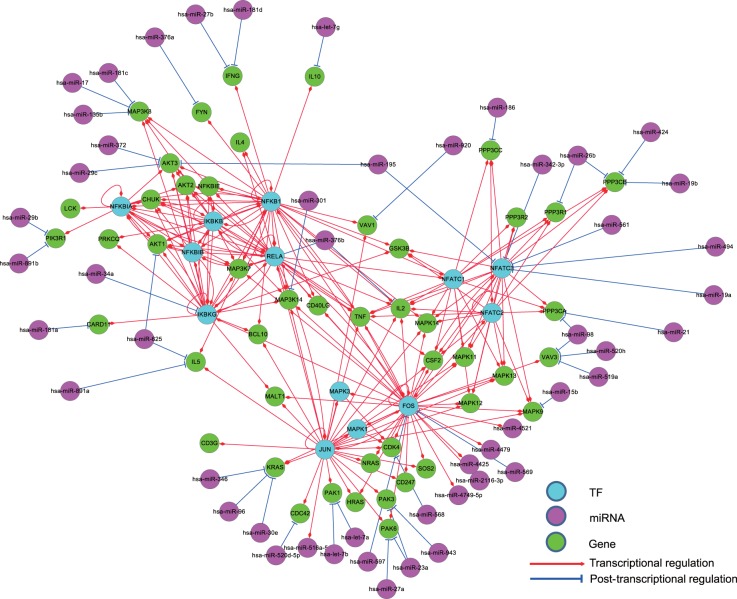
The regulatory relationships of a KEGG gene set for the human T cell receptor signaling pathway in RegNetwork. TF, miRNA and gene are in different colors and the transcriptional and post-transcriptional interplays are shown in red and blue, respectively.

Different from the existing regulatory relationship databases such as TRED (47), RegNetwork contains both the transcriptional and post-transcriptional regulatory interactions, which allows us to investigate more complex interplays between regulators (i.e. TF and miRNA) and their target genes. Figure 6 illustrates the collected interactions from a KEGG gene set involved in the T cell signaling pathway in human, where the post-transcriptional regulatory relationships are drawn as blue lines. Since network motif is an important local property and functional block of complex network, here we identify the three-node network motifs (‘TF-miRNA-gene’) in the established regulatory networks. Figure 6 clearly suggests the combinatorial control of gene expressions mediated by TFs and miRNAs simultaneously. For instance, visually we can identify several network motifs in Figure 6, such as ‘FOS’-‘hsa-miR-569’-‘MAPK12’ and ‘JUN’-‘hsa-let-7a’-‘PAK1’, which are believed to be the major network building components and functional blocks in regulatory networks (48). By this simple example, we show that RegNetwork is a useful tool for querying the knowledge-based combinatorial regulatory relationships in both transcription and post-transcription. Actually, using the network motif detection algorithm, FANDOM (49), we can identify all the three-node motifs ‘TF-miRNA-gene’ in the human and mouse regulatory networks, respectively. Table 4 lists their occurrence frequencies and the statistical significance in the form of Z-scores. Ten types of ‘TF-miRNA-gene’ motifs are identified in the two networks. For each type of the motifs, the Z-score is calculated as the difference of its actual occurrence frequency and the average of its occurrence frequencies in 100 random networks of the same node-size, normalized by the standard deviation of these random occurrence frequencies, and the motifs with a Z-score higher than 2 are regarded as significantly enriched according to FANDOM (49). As shown in Table 4, ‘M1’, ‘M2’, ‘M3’, ‘M5’ and ‘M7’ are enriched in both human and mouse regulatory networks; ‘M10’ are enriched in one of them, and motifs ‘M4’, ‘M6’, ‘M8’ and ‘M9’ are not enriched in either of them. The enrichment of different types of motifs suggests a major topological and statistical change in the local network structures, which is of significant scientific interest and a promising research approach for understanding context-specific (e.g. certain disease) regulatory machineries (31).

**Table 4.** The three-node network motifs ‘TF–miRNA–gene’ in human and mouse regulatory networks

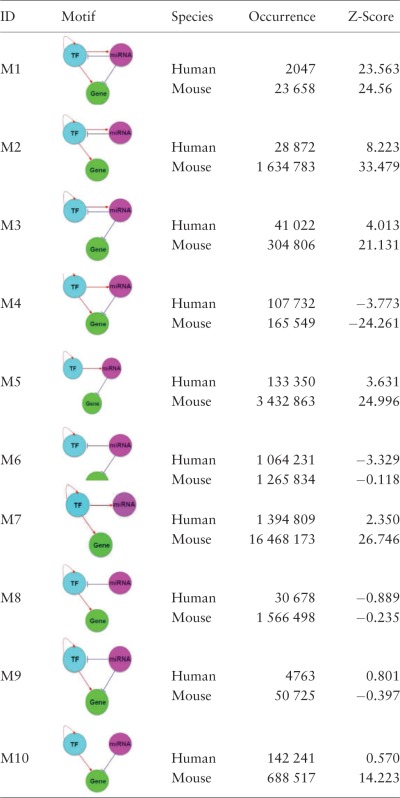

The motifs are ranked by the absolute Z-Scores of network motifs in human. The higher the Z-Score, the more enriched is a motif (threshold is 2 as suggested in FANDOM (47)).

## Conclusion

In this article, we developed a database, RegNetwork, of the knowledge-based genome-wide regulatory networks in human and mouse by integrating various data sources. A comprehensive set of interplays among TFs, miRNAs and target genes were collected and reorganized for public access. The established regulatory networks from RegNetwork provide genome-wide regulatory interactions, which lay an initial foundation and establish a prior background network to identify or verify molecular and functional regulations in pathways or subnetworks corresponding to different phenotypes. Also, combined with high-throughput expression data under specific physiological and developmental conditions (e.g. viral infection), one can identify differential subnetworks and pathways from the background networks in RegNetwork, which will lead to novel and interesting insights into regulatory mechanisms in context-specific processes.

At the time when the current version of RegNetwork was developed, the ENCODE project published thousands of regulatory interactions in human inferred from high-throughput datasets (50), which contains 162 100 regulatory relationships among 119 TFs, 736 miRNAs and 15 131 genes. Most of the TFs, miRNAs and genes in ENCODE (96.5% of the regulators and 89.9% of the targets) are already included in our database. We will continue to track and regularly integrate the ENCODE regulatory relationships into our database. We also recognize the usefulness of text mining tools to identify and curate the regulatory relationships from literature, which is another direction to extend the RegNetwork. We also plan to extend the RegNetwork to include additional information such as the experimental conditions and original references for each of the regulatory relationships that are derived from. We will also extend the RegNetwork to include other organisms, such as *Rattus norvegicus* (rat), *Drosophila melanogaster* (fruit fly), *Caenorhabditis elegans* (worm), *Escherichia coli* (*E. coli*) and *Saccharomyces cerevisiae* (yeast).

## Funding

National Natural Science Foundation of China (NSFC) (Grant Nos. 61572287 and 61533011 to Z.P.L.); the Shandong Provincial Natural Science Foundation of China (Grant No. ZR2015FQ001 to Z.P.L.); the Fundamental Research Funds of Shandong University (Grant No. 2014TB006 to Z.P.L); the Scientific Research Foundation for the Returned Overseas Chinese Scholars, Ministry of Education of China (to Z.P.L.); University of Rochester Center for Biodefense Immune Modeling Grant (NIH/NIAID) (HHSN272201000055C to H.W.); University of Rochester Center for AIDS Research Grant (NIH/NIAID) (P30AI078498 to H.W); NIH Grant (R01GM100788 to H.M.). Funding for open access charge: Dr. Hulin Wu's start-up fund.

*Conflict of interest*. None declared.
